# A dynamic systems perspective towards executive function development: Susceptibility at both ends for inhibitory control

**DOI:** 10.1017/S0954579422000037

**Published:** 2022-03-02

**Authors:** Qiong Wu, Karina Jalapa, Soo Jin Han, Dania Tawfiq, Ming Cui

**Affiliations:** Department of Human Development & Family Science, College of Health and Human Sciences, Florida State University. Tallahassee, FL, USA

**Keywords:** dynamic systems perspective, executive function, infant negative reactivity, maternal sensitivity, optimal arousal perspective

## Abstract

In light of the dynamic systems perspective, the current study expanded existing literature by examining the moderating effect of maternal sensitivity on the quadratic association between infant negative reactivity and future executive function development. Using a longitudinal, multimethod design, we addressed executive function development among preschoolers. This study utilized data from the Family Life Project (*N* = 1292). Infant negative reactivity at 6 months, maternal sensitivity across first 3 years, and executive functions during preschool age were observational assessed. A path model with moderation analyses revealed a U-shaped quadratic association between infant negative reactivity and preschoolers’ inhibitory control, only when maternal sensitivity was high. The results suggest that maternal sensitivity may assist infants with both low and high, but not moderate, levels of negative reactivity towards better executive function development. Findings support the ongoing nonlinear person-environment interplay during early years of life.

Executive function refers to an individual’s cognitive abilities to achieve goal-directed behaviors (Anderson, 2002). Executive function in childhood has been associated with higher levels of academic and social competence, and lower levels of emotional and behavioral problems (Becker et al., 2014; Garon et al., 2008; White et al., 2011). Early development of executive function can be influenced by both one’s temperament (Rothbart et al., 2011) and the caregiving environment (Gueron-Sela et al., 2017; Wu et al., 2021). Multiple theories on an integrated self-regulation framework (e.g., Blair & Raver, 2015; Bridgett et al., 2015; Feldman, 2009) point to the potential to investigate the influence of early temperamental negative reactivity on executive function development, but evidence examining such an association remains insufficient. Embracing a dynamic systems perspective (Lewis, 2005; Spencer et al., 2011), the current study investigated the prospective relations between infant temperamental negative reactivity and the development of different executive function components in early and middle childhood, while examining the potential moderating effect of maternal sensitivity.

Executive function has been mostly conceptualized as a unitary construct consisting of several interconnected but separate elements. During early childhood, young children increase in their *inhibitory control* (i.e., the capability to suppress a dominant response to allow for a subdominant response), *attention flexibility* (i.e., the flexibility to shift attention between multiple mental tasks), and *working memory* (i.e., manipulating and updating information retained in mind for a period of time; Fuhs et al., 2014; Garon et al., 2008; Gerstadt et al., 1994; Munakata et al., 2012; Willoughby et al., 2010; Wolfe & Bell, 2007). Some researchers argue that the interrelated components of executive function have different precursors (Buss et al., 2011; Ku et al., 2019; Noble et al., 2005; Sarsour et al., 2011) and can introduce distinct cognitive and emotional outcomes among preschoolers and school-aged children (e.g., Becker et al., 2014; Blakey et al., 2016; Clark et al., 2010; White et al., 2011). Biobehavioral models also suggest that unique neural processes underlie each dimension and can manifest as different behavioral indicators of executive function components (e.g., Aron, 2008; Kieffer et al., 2013). As such, researchers call for an examination of executive function as both interconnected and distinct components (Baggetta & Alexander, 2016).

## A dynamic systems perspective of executive function development

A dynamic systems perspective (Lewis, 2005) of child development presumes that systems are self-organizing, and that lower-order functions serve as important building blocks for higher-level regulatory functions (Feldman, 2009). It further assumes *nonlinear* and *time-dependent interactions* among lower-level elements in integration into higher-level regulatory entities (Spencer et al., 2011). In particular, the system has an intrinsic tendency to form patterns by uniting lower-level processes (i.e., bottom-up processes such as emotional reactivity) into higher-rank functions (e.g., top-down self-regulatory functions), and this formation process can follow nonlinear, such as curvilinear, associations. Theories and empirical evidence have suggested emotional reactivity in infancy as an essential building block for future executive function development (Feldman, 2009; Ursache et al., 2013). Indeed, a conventional, unidirectionally linear perspective may suggest that a high level of emotional reactivity could be indicative of poor regulatory functions. However, the complexity of the dynamic, systemic processes usually embraces nonlinear thinking.

The nonlinear strength of emotion arousal was first documented in the classic psychological experiment, in which researchers found an inverted U-shaped association between arousal and learning (Yerkes & Dodson, 1908). That is, the moderate arousal level is optimal for performing cognitive tasks. This notion has been subsequently supported by neurobiological evidence that an increase in stress hormone levels shows an inverted-U, quadratic association to synaptic activity in prefrontal cortex to perform executive cognitive tasks (Ramos & Arnsten, 2007). As such, researchers posit that an *optimal arousal level* exists for cognitive regulation. In contrast, too much negative reactivity may override an individual’s learning processes, whereas too little negative reactivity may provide few chances for an individual to practice and to refine their regulation skills (Geeraerts et al., 2020; Ursache et al., 2013).

In support of the optimal arousal perspective, a moderate level of fussing during infancy was associated with the highest level of inhibitory control and attention shifting among 4-year-olds (Geeraerts et al., 2020). Moderate fluctuations in salivate cortisol levels (a biomarker of stress) between 6 months and 4 years combined with low cortisol levels predicted better preschool executive function than highly stable or highly variant cortisol levels (Blair et al., 2017). A moderate level of vagal tone (an indicator of autonomic functioning) in early childhood predicted greater prosocial behaviors in middle childhood (Miller et al., 2017). A moderate level of vagal withdrawal (physiological regulation of stress) was associated with higher working memory and inhibitory control among preschoolers (Marcovitch et al., 2010). The preliminary evidence supports further investigations into executive function development in both early and middle childhood regarding an optimal arousal perspective.

## Maternal sensitivity as the environmental context

A dynamic systems perspective of child development also suggests that the development needs to be understood in context, where the developing mechanisms constantly absorb and consolidate from external influences until they become inseparable (i.e., the “organism in context” as an inseparable study unit; Spencer et al., 2011). This *constant consolidation* process induces small, quantitative changes which then assemble into large, qualitative different changes over time. Moreover, learning happens from multiple interacting components on the basis of both the self-organizing mechanism and the environmental context, which supports a broader view of personal-environmental interplay but in a more time-dependent manner (Spencer et al., 2011). One of the commonly examined contextual influences of executive function development is parental sensitivity (Bernier et al., 2010; Gueron-Sela et al., 2017). Sensitive parenting refers to parenting behaviors that show understanding, attention, and contingent responses towards children’s emotional cues (Ainsworth et al., 1978). Sensitive parenting during early childhood is key to the emergence and continued development of executive function (Bernier et al., 2010; Geeraerts et al., 2020; Gueron-Sela et al., 2017). The early childhood years are a susceptive period for parental sensitivity to contribute to the maturation of amygdala-prefrontal circuitry - the brain networks that are important to cognitive control (Gee et al., 2014; Wang et al., 2019). For example, sensitive parenting aids toddlers in the process of gaining attention shifting abilities by contingent support towards regulatory capacities (Crockenberg & Leerkes, 2004). Sensitive parenting also increases preschoolers’ inhibitory control by providing support towards internalizing rules (Bernier et al., 2010; Geeraerts et al., 2021).

Theories and evidence regarding possible nonlinear person-environment interplay remain insufficient. Most current theories are built on the basis of linear thinking, and they do not fully agree on the levels of infant negativity that are most susceptible to environmental influences. For example, the diathesis-stress model emphasizes that children with higher negativity experience more risk when the environment is not supportive (aka the “dual-risk” model; Ingram & Luxton, 2005). In contrast, the differential susceptibility model suggests that these children may also uniquely benefit more in a highly supportive environment, compared to their less-negative peers (Roisman et al., 2012).

Current research suggests a possibility that under high maternal sensitive parenting, children with lower and higher negative reactivity can both benefit. *Highly* negative infants showed better self-regulation (effortful control and compliance) in toddlerhood when in responsive mother-child relationships (Gueron-Sela et al., 2017; Kim & Kochanska, 2012; Poehlmann et al., 2011). On the other end, infants with *lower* reactivity were also prone to maternal sensitivity in reducing affect dysregulation (Leerkes et al., 2009). Geeraerts et al. (2020) found that infants with *moderate* levels of fussing were more susceptible to the effect of maternal sensitivity in the development of inhibition and attention shifting, showing an inverted U-shaped association. Other studies showed nonsignificant linear associations between child negative reactivity and executive function (e.g., Gartstein et al., 2013; Ku et al., 2019), possibly due to a lack of examination of quadratic effects. Given maternal sensitivity is typically more influential towards those with the highest need of assistance (Gueron-Sela et al., 2017; Kim & Kochanska, 2012), it is reasonable to expect that children with *both* lower and higher negative reactivity benefit more from it, as they may encounter greater challenges in executive function development (Ursache et al., 2013). However, intriguing questions remain whether different executive function components are similarly susceptible to such a possible nonlinear moderation association.

## The current study

In light of the dynamic systems perspective (Lewis, 2005; Spencer et al., 2011), the current study expanded existing literature by examining the moderating effect of maternal sensitivity on the quadratic effect of infant negative reactivity on future executive function development. We focused on the preschool age, a key developmental stage for gains in executive functions such as *working memory*, *inhibitory control*, and *attention flexibility*. We investigated maternal sensitive parenting behaviors over early childhood to further our understanding of the constant consolidation process (Spencer et al., 2011). This study utilized a unique sample of low-income, rural families, which typically experience risks in executive function development (Sarsour et al., 2011). We included important covariates to children’s executive function development, such as sex, race, and family income.

We tested quadratic associations between infant negative reactivity and later executive function development. We further examined whether the strength of the quadratic association differed depending on the levels of maternal sensitivity. We expected a stronger U-shaped quadratic association between infant negative reactivity and executive function when maternal sensitivity was high (versus low), such that infants with both lower and higher negative reactivity would show higher executive function under high maternal sensitivity. As few studies examined different executive function components in such proposed quadratic associations, we examined executive function as separate components, and our hypothesis regarding which executive function would be susceptible to infant negative reactivity remained exploratory.

## Method

### Participants

Data included 1292 children within their family contexts from the Family Life Project (FLP; Vernon-Feagans et al., 2013). Families who participated in the FLP were recruited from hospitals in geographical areas with high poverty rates within the United States. The FLP used a stratified sampling method to oversample low-income and African American families. Mothers’ mean age was 26.4 years (*SD* = 6.0) when their infants were 6 months old. Child sex in the sample was balanced (50.9% boys). The sample included 58.8% of mothers who reported to be White, 40.7% African American, and 0.5% other races. Nearly half of the mothers (48.6%) from the sample were married and 40.9% of the mothers were employed at the time of enrollment. Mothers reported having less than a high school education (19.8%), having a GED/high school certificate (39.2%), having some college education (27.2%), or having a college degree and above (13.8%).

### Procedures

When the children were 6-, 15-, 24-, and 35-month old, trained research assistants visited the families’ homes for data collection. Mothers were asked to report demographic information at the 6-month visit, when infant negative reactivity was assessed. Maternal sensitivity was assessed four times when children were 6-, 15-, 24-, and 35-month old. Preschoolers’ executive function was measured during the 35-month visit.

### Measures

*Infant negative reactivity* was observed at 6 months old through three tasks. In the *Mask Task* (Goldsmith & Rothbart, 1996), the research assistant sat by the infant and wore four scary masks, each for 10 s. The research assistant called the infant’s name each time the mask changed while moving their head slowly from side to side and then leaned towards the infant. The *Arm Restraint Task* (Stifter & Fox, 1990) was also administered to observe emotion regulation. For this task, a research assistant stood behind the infant and gently held the infant’s arms down for 2 min before releasing them for 1 min. During the free minute, the research assistant stayed hidden behind the infant while the infant regulated themselves before maternal comfort. For the *Barrier Task* (Goldsmith & Rothbart, 1996), the infant was given an appealing toy to interact with for 30 s. When the time was up, the research assistant gently removed the toy and placed it behind a Plexiglass barrier in front of the infant for 30 s. This pattern was repeated for a total of three times. These tasks were videotaped and coded for the infant’s facial affect and vocalizations (e.g., screams, frowning, wide squared mouth, tears, facial color change), using a second-to second coding scheme (Stifter & Braungart, 1995). The percent of time showing negative expression was used as the indicator of negative reactivity. Inter-rater reliability was high with Kappa’s between .85 and .95 across the three tasks (15% of the videos were double-coded).

*Maternal sensitivity* was observed through a 10-min free-play interaction task with the infant when the infant was 6- and 15-months old. When the child was 24- and 35-months old, the child and mother were asked to complete a jigsaw puzzle. Once the first puzzle was completed, two more complicated puzzles were presented. The tasks were also videotaped and later coded for maternal parenting (Cox et al., 1999; NICHD Early Child Care Research Network, 1999). Seven maternal behaviors were coded: *sensitivity* (being responsive to the child’s social gestures and negative affect such as cries and frets), *intrusiveness* (mother-centered interaction), *detachment* (being emotionally uninvolved and unaware of child’s needs, and being distant without facilitating the child’s involvement with toys or people), *positive regard* (positive affect or verbalizations towards the child), *negative regard* (negative expressions and hostility towards the child), *animation* (excitement, level of energy), and *stimulation of development* (scaffolding of activities to foster or simulate child’s play at a developmental appropriate level). Ratings were made on a 1–5 scale (1 = “*not at all characteristic*” to 5 = “*highly characteristic*”). Inter-rater reliability (intraclass correlation coefficients) between coders ranged from .74 to .89 for all codes, with 30% of the cases double-coded.

To obtain maternal sensitivity scores, principal component analyses with oblique rotation were conducted at each assessment. Ratings for sensitivity, detachment (reversed), stimulation of development, positive regard, and animation loaded on one dimension, explaining a significant amount of variance (50.5% at 6 months, 53.7% at 15 months, 56.1% at 24 months, and 48.2% at 35 months). A mean score of these five subscales was generated as the mother’s level of sensitivity. The mean scores of maternal sensitivity were 2.90 (*SD* = 0.79), 2.79 (*SD* = 0.80), 2.89 (*SD* = 0.81), and 2.88 (*SD* = 0.72) for 6, 15, 24, and 35 months (on the scale of 1–5), respectively. Thus, mothers with higher sensitivity would be mostly moderately, or at least somewhat characteristic on behavioral indicators such as attending to the child’s affective communications and gestures, being involved with the child, appearing to be happy and give positive comments towards the child, showing excitement, and scaffolding the child’s play. In contrast, mothers with lower sensitivity would be minimally characteristic on these behavioral indicators. Scores at different timepoints are highly correlated with each other (*r* ≥ .55, *p* < .001), and thus were standardized and then averaged into a final score of mean maternal sensitivity over time, similar to previous studies (e.g., Adi-Japha & Klein, 2009; Kochanska & Kim, 2013; Miner & Clarke-Stewart, 2008; Wu et al., 2021). A higher score indicated higher sensitivity. This score was highly correlated with scores across four assessment timepoints (*r* ≥ .83, *p* < .001).

Preschoolers’ executive functions were measured by a series of tasks during the 35-month assessment. In particular, *working memory* was measured through the *Working Memory Span* task (4 items; Engle et al., 1999). For this task, the child was presented with a simple line drawing of an animal and colored dot placed inside a house. The child was then asked to name the animal and then to name the color on the page. The research assistant then displayed an image of the same house but this time the house was empty. The child was then asked to recall what animal was previously in the house.

Preschoolers’ *inhibitory control* was assessed with three different tasks, with the first being the *Animal Go/No Go* task (Durston et al., 2002). In this task, children were given a button to press each time the research assistant showed a picture of an animal (“go” trial). If the animal shown was a pig, the child was instructed to refrain from pressing the button (“no-go” trial). There were varying numbers of “go” trials before each “no-go” trail, with a total score from the 7 no-go trials calculated. For the *Spatial Conflict* task (Gerardi-Caulton, 2000), the child was given two side-by-side buttons. The child was shown an arrow which prompted the child to touch the button that corresponded to the direction that the arrow pointed towards. The child was given a task familiarizing phase where the arrow was always in the center for the first 8 trials. After that, the arrows pointed either to the left side or right side for trial 9–22, to build prepotency to touch to corresponding button. For the remaining trials (23–35), the arrows began to appear in a random order and contralaterally (left-pointing arrows on the right and right-pointing arrows on the left) which requires inhibitory control to suppress the previously established prepotent response. The contralateral trials were used towards scoring inhibitory control. The last task was the *Silly Sounds Stroop* task (17 items; Gerstadt et al., 1994) where children were shown pictures of either a cat or a dog and instructed to meow at the dog photo and bark at the cat photo.

Preschoolers’ *attention shifting* was measured by the *Something’s the Same* task (14 items; Jacques & Zelazo, 2001). For this task, the child was presented with a page that had two items similar in shape, size, or color. The research assistant and child then discussed what made the items similar. Following the discussion, the research assistant turned to a different page where an additional item was added to the previously seen items. The third item was similar to one of the previous items in a different dimension. The child was then asked to identify which of the two original items was similar to the third item.

These executive function measures were scored using item response theory models, which utilized children’s response accuracy of each item to generate an expected a posteriori (EAP) score for each task. The EAP estimation belongs to a larger school of thoughts to estimate one’s latent trait (i.e., multidimensional latent trait models). The basic assumption of EAP scores is that every individual may succeed or fail on any item despite the item difficulty of this person’s ability, thus rather than scoring the accuracy directly, the EAP scores present one’s likelihood to succeed in this task. The EAP scores can account for previous data (e.g., the item’s difficult of the person’s ability) to generate a calibrated score for this person, derived from Bayesian statistical principles. The EAP scores are suitable to process dichotomous or polytomous to indicate a latent trait score for an individual by accounting for the expected value of the posterior probability distribution (Willoughby et al., 2011). This method was recommended to use in item response theory models, over conventional methods such as using this person’s sum of item accuracy scores directly (Nicewander & Schulz, 2015). For working memory and attention shifting, the EAP scores of the task were directly used. For inhibitory control, the mean score of the three tasks were used. A negative EAP score indicated that the individual had less than a half chance to succeed in the task, and a positive EAP score indicated that the individual had more than a half chance to succeed. Higher scores indicated better executive function. More details about the tasks, the EAP scores, the psychometric properties and the longitudinal measurement invariance of the executive function assessments, can be found in previous studies (e.g., Willoughby et al., 2010, 2012).

*Covariates* in this study were the child’s sex (1 = *male*, 2 = *female*), race (0 = *African American*, 1 = *White*), the family’s residential state (0 = *North Carolina*, 1 = *Pennsylvania*), and family income-to-needs ratio (as a proximity of family socioeconomic status, calculated by dividing total family income by the poverty threshold for the family size) at 6 months. These variables were included due to their theoretical or empirical relevance to main study variables. For example, girls are likely to have better executive function scores than boys in early childhood (Mileva-Seitz et al., 2015). Minority children in lower income family may disproportionally experience difficulties in executive function development (Rhoades et al., 2011). Residential state was included as a control for the effect of recruitment sites, as there were significantly fewer African American families in the Pennsylvania site than the North Carolina site (Vernon-Feagans et al., 2013).

### Analytic strategies

Analyses were conducted using the lavaan package (Rosseel, 2012) in R (R Core Team, 2014) using the path model approach. The path model approach allows for simultaneous estimations of covariances (i.e., common components) among executive function elements as well as predictors towards variances that are unique to each element. Little’s MCAR test (Little, 1988) revealed that data were not missing completely at random, χ^2^(81) = 141.00, *p* < .001. Low income-to-needs ratio was associated with the missingness of infant negative reactivity at 6 months, *t*(213.7) = 2.4, *p* = .02. Low income-to-needs ratio, low maternal sensitivity, and low infant negative reactivity at 6 months were also related to the missingness of executive function variables at 35 months (*t*s ≥ 1.9, *p*s ≤ .05). As missingness of data could be explained by other study variables, the data missing pattern was likely random. Thus, full information likelihood estimation was used to reduce bias for missing data estimation (Enders & Bandalos, 2001), with robust standard errors (MLR) to handle potential skewness.

The path model was set up by including infant negative reactivity, maternal sensitivity, the quadratic term of infant negative reactivity, the quadratic term of infant negative reactivity × maternal sensitivity, as well as the covariates (child sex, race, family residential state, and income-to-needs ratio), to predict three separate executive function components (working memory, inhibitory control, and attention flexibility). Covariances among executive function components were estimated. This analysis attempted to reveal that for an infant starting with certain negative reactivity levels at 6 months, what their executive function might look like if continuously exposed to high or low maternal sensitivity over 2.5 years. The model fit of the path model was evaluated by the root mean squared error of approximation (RMSEA), comparative fit index (CFI) and the Tucker–Lewis Index (TLI), with a RMSEA of 0–.05 and CFI/TLI of .95–1.00 indicating good fit and a RMSEA of .05–.08 and CFI/TLI of .90–.95 showing acceptable fit (Hu & Bentler, 1995). In illustrating the interactions, simple slope tests were conducted at low and high levels of maternal sensitivity, centered at one standard deviation below the mean and above the mean, respectively.

## Results

Descriptive statistics of study variables are presented in Table 1. On a bivariate level, maternal sensitivity over early childhood was positively related to higher levels of child working memory, inhibitory control, and attention shifting at 35 months. Infant negative reactivity was related to higher child attention shifting at 35 months in a linear pattern. Girls had higher inhibitory control. White children and children in higher-income families had higher maternal sensitivity and higher executive function scores. Children in the Pennsylvania site were more likely to be White and to live in higher-income families, and relatedly they also had higher maternal sensitivity and higher executive function scores.

**Figure 1.**
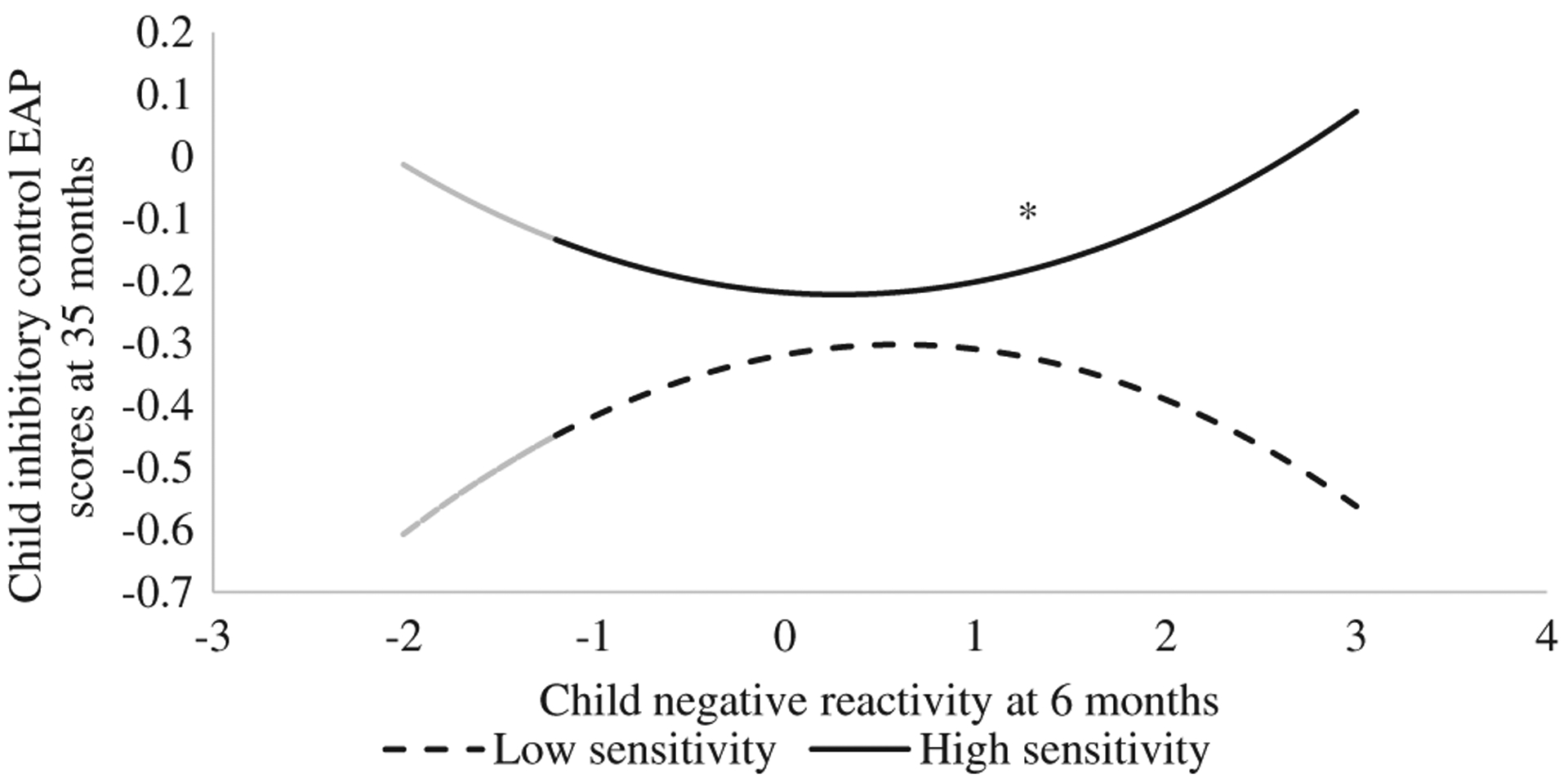
The interaction between observed infant negative reactivity at 6 months and maternal sensitivity predicting child inhibitory control at 35 months. Every unit of the *x*-axis indicates one standard deviation (*SD*), and zero indicate the mean of infant negative reactivity. The actual variable range is −1.15–4.07 *SD*. Simple slopes below −1.15 *SD* is presented in gray color due to no available data in this region. *indicates the statistically significant simple slope.

The path model was saturated, thus showing a perfect model fit, χ^2^(0) = 0.000, CFI = 1.000, TLI = 1.000, RMSEA = .000 (90% CI [.000, .000]). As shown in Table 2, maternal sensitivity over early childhood predicted higher working memory (*B* = 1.06, *SE* = 0.41, *t* = 2.60, *p* = .009) and attention shifting (*B* = 1.37, *SE* = 0.43, *t* = 3.17, *p* = .002) at 35 months, whereas infant negative reactivity at 6 months predicted higher attention shifting (*B* = 0.34, *SE* = 0.16, *t* = 2.11, *p* = .04). Additionally, an interaction between the quadratic term of infant negative reactivity and maternal sensitivity emerged in predicting preschoolers’ *inhibitory control* (Figure 1). The U-shaped quadratic effect of infant negative reactivity on preschoolers’ inhibitory control was only significant when maternal sensitivity was high (*B* = 0.10, *SE* = 0.05, *t* = 2.19, *p* = .03). When maternal sensitivity was low, infant negative reactivity did not predict inhibitory control in quadratic (*B* = −0.11, *SE* = 0.08, *t* = −1.43, *p* = .15) or linear formats (*B* = 0.27, *SE* = 0.23, *t* = 1.17, *p* = .24). The covariance of attention shifting and inhibitory control was correlated (*B* = 6.87, *SE* = 1.79, *t* = 3.84, *p* < .001), but not those between attention shifting and working memory (*B* = 2.48, *SE* = 1.64, *t* = 1.52, *p* = .13) or between inhibitory control and working memory (*B* = 1.74, *SE* = 1.46, *t* = 1.20, *p* = .23).

In the supplemental material, we performed the same analysis but with maternal sensitivity at 6 months alone, to better understand the consolidation process during early childhood. Results mirrored the findings in the main manuscript, especially the interaction pattern. Please refer to the supplemental material for analytic details and finding comparisons.

## Discussion

The current study adopted a dynamic systems perspective (Lewis, 2005; Spencer et al., 2011) in the investigation of the quadratic associations between infant negative reactivity and child executive function development. Using a longitudinal, multimethod design, findings supported maternal sensitivity as a moderator in the associations between infant negative reactivity and later executive function development. This study has significant implications to future research and intervention efforts.

### Infant negative reactivity and executive function among preschoolers

As expected, we identified a U-shaped quadratic association between infants’ negative reactivity and preschoolers’ inhibitory control, only when maternal sensitivity was high. Specifically, under high maternal sensitivity, infants with both higher and lower negativity had higher inhibitory control, compared to infants with moderate negativity. It is likely that for infants with lower levels of negativity, it is easy to gain inhibition with contingent parenting and rule internalization (Kim & Kochanska, 2012). On the other hand, high levels of negativity may provide infants with opportunities where sensitive parenting may assist with managing emotions and impulses, thus promoting inhibitory control (Geeraerts et al., 2021; Gueron-Sela et al., 2017). In contrast, infants with moderate negative reactivity may not receive such beneficial effects from maternal sensitivity as they are less “at risk.” They may exhibit lower executive functions in a lower resourced context where maternal sensitivity is especially crucial for self-regulation development (Wu et al., 2021), when compared to their peers with higher or lower negative reactivity who also benefit greatly from maternal assistance. The quadratic effect of infant negative reactivity is prominent with preschoolers’ inhibitory control, as this executive function component particularly requires managing arousals and impulses that benefits from parental support (Geeraerts et al., 2021). These findings are consistent with the dynamic systems perspective which points to the nonlinear associations from lower-level components in forming higher, self-regulatory functions, as well as the constant consolidation process from the organism-environment interaction (Spencer et al., 2011).

Additionally, infant negativity predicted higher attention shifting among preschoolers. This finding is unexpected. However, a higher negative reactivity can provide young children to practice necessary opportunities to regulate emotional arousals (Ursache et al., 2013). One of the commonly used emotion regulation skills by young children is attention control, or actively reengage attention towards less emotionally eliciting events (Crockenberg & Leerkes, 2004; Wu et al., 2020). As such, it is possible that highly negative infants can practice their attention shifting skills when they need to reengage attention to regulate negative arousals. Studies also indicated that toddlers high in negative reactivity showed higher attention shifting skills during emotional-eliciting events (e.g., Kiel et al., 2020; Wu & Gazelle, 2021), supporting such claims.

We did not find an association between negative reactivity in infancy and working memory among preschoolers. This nonsignificant finding was similarly reported by previous studies (e.g., Wolfe & Bell, 2007). It is likely that the ability to manage negative arousals do not facilitate nor hinder early memory development (Wolfe & Bell, 2007).

Our findings may appear to be different from those in Geeraerts et al.’s (2020), which showed an inverted U-shaped association between infant fussing and preschoolers’ executive functions under high maternal sensitivity. One of the possible explanations is that Geeraerts et al. (2020) investigated inhibitory control and attention shifting together, which may not reveal the unique variations of each of the executive function components. Indeed, we found that infant negative reactivity was differentially associated with both inhibitory control and attention shifting among preschoolers. Second, Geeraerts et al. (2020) assessed infant fussing as an indicator of low-intensity negative reactivity, whereas the current study only included infant general negative reactivity due to the available data. Additionally, Geeraerts et al. (2020) utilized a sample that was predominantly White (93%), from intact families (99%), middle-class and above, and highly educated (all mothers had finished high school). In comparison, this study included a sample of low-income, rural children (with a high proportion of African Americans) who may experience lower levels of maternal sensitivity compared to those from more affluent backgrounds (Wu et al., 2021). As such, the effect of maternal sensitivity may be more salient for children at either ends of negative reactivity where children may experience unique risks. Finally, our study was consistent with Geeraerts et al. (2020) in the findings regarding low levels of maternal sensitivity under which infant negative reactivity was not associated with preschoolers’ executive function. It seems that high maternal sensitivity facilitates lower bottom-up processes to form higher self-regulatory functions, but low sensitivity does not (Wu et al., 2021).

### Theoretical integration

Taken together, findings from both studies provide robust evidence on the optimal infant negative reactivity levels to facilitate the emergence and continued growth of multiple components of executive functions. Several theoretical perspectives have touched on this topic, such as the diathesis-stress model (Ingram & Luxton, 2005), the differential susceptible perspective (Roisman et al., 2012), and the integrated self-regulation framework (Bridgett et al., 2015). Our findings extended these models by uniquely revealing that infants of both lower and higher negativity can benefit from sensitive parenting, thus displaying higher executive function levels, as compared to infants with moderate negativity levels. This finding extends the optimal arousal perspective by indicating infants with higher needs in assisting regulation (e.g., having higher or lower negativity) are also more susceptible to maternal support, emphasizing on the consolidation process during development from external forces (e.g., sensitive parenting).

Our study supports the dynamic systems perspective (Lewis, 2005; Spencer et al., 2011) and furthers the current, predominantly linear investigations on the association between negative reactivity and executive function by providing a nonlinear thinking of the multiple components in organizing towards a unified regulatory function. This study taps particularly into inquiries about the ongoing nonlinear contributions (rather than linear, unidirectional associations) across cognitive and emotion regulation functions, according to a dynamic systems perspective (Lewis, 2005; Spencer et al., 2011). This study further reveals that each executive function may have unique precursors, and these precursors can be both internal (e.g., the child’s own characteristics) or external (e.g., caregiver and environmental influences), supporting a discrete approach in understanding executive function development (Baggetta & Alexander, 2016). The findings also point to the constant consolidation process during early childhood (Spencer et al., 2011), which supports the important ongoing person-environment interplay and reveal key pathways for maternal sensitivity to facilitate self-regulation development. Finally, this study also has implications for self-regulation development in a lower-resourced context.

### Strengths, limitations, and implications

This study has several noticeable strengths. This study employed a longitudinal sample to provide robust associations between infant negativity and various types of child executive functions in a key developmental stage. This study utilized a multimethod approach in assessing key study variables, including both laboratory observation and maternal report of child temperament, as well as children’s own responses to the executive function tasks. Additionally, this study included a low-income sample that was under-studied but tended to experience risks in executive function development (Sarsour et al., 2011). Maternal sensitivity was repeatedly assessed across 2.5 years to obtain an overall sensitivity score over early childhood, which provides us with knowledge about the caregiving environment over time and better captures the constant consolidation process by children during early childhood. Additionally, this measure decreases likelihood of time-varying (e.g., a stressful time period of marriage) or situational effects (e.g., a bad day) over measurement at a single time point, thus reducing possible assessment errors.

Several limitations need to be noticed when interpreting the study findings. First, this study oversampled low-income, rural families, which, despite its unique strengths, might limit the generalization of the current findings towards families with higher income. Second, this study only assessed maternal sensitivity, therefore findings may not generalize to other caregivers such as fathers. Third, due to our observational assessment on child negativity, the range of this variable, especially in the lower negativity direction, was relatively limited (as low as 1.15 *SD* below mean; Figure 1). This might have limited our understanding of infants with lower negativity. Further studies should include a variety of infant negativity measures (e.g., parental report, laboratory tasks, observer ratings) to obtain a wider range of infant negativity to replicate such findings. Additionally, we observed a trend of a curvilinear effect when maternal sensitivity was low, which had a similar size to that under high maternal sensitivity but was not statistically significant. Although neither our study nor Geeraerts et al. (2020) found significant curvilinear effects under low maternal sensitivity, this finding needs to be interpreted with caution and be replicated in future studies. Finally, there can exist multiple mother-child exchanges during development, and mothers may adjust their parenting based on children’s negativity or executive function levels (e.g., Kim & Kochanska, 2012). Thus, future studies should take this parent-child mutual influence into account when investigating the multifaceted associations among infant negative reactivity, sensitive parenting, and executive function development.

In conclusion, the current study indicates that the early influence of child negative reactivity on executive function development is a nonlinear process, and maternal sensitivity aids in this process by channeling infants with higher and lower negativity towards better cognitive outcomes. The effects of early sensitive parenting can create a long-lasting secure base for children to develop their cognitive abilities in early and middle childhood. Findings of this study provides insight into facilitating support towards continued sensitive parenting over early childhood and reducing risks factors that may compromise maternal sensitivity (e.g., stress, mental health concerns). Together, a more complete understanding of factors contributing to children’s executive function development will promote adaptive children’s academic, emotional, interpersonal, and physical functioning in early and middle childhood, which lays a life-long foundation of success and happiness for children.

## Supplementary Material

1

## Figures and Tables

**Table 1.** Descriptive statistics and bivariate correlations of study variables

	1	2	3	4	5	6	7	8		9
1. Child sex	–									
2. Child race	−.02	–								
3. State	−.08**	.60**	–							
4. Income-to-needs ratio	−.05	.38**	.24**	–						
5. Maternal sensitivity	.02	.41**	.29**	.45**	–					
6. Infant negative reactivity 6 months	.08*	.03	−.03	.07*	.06*	–				
7. Child working memory 35 months	.06	.30**	.25**	.25**	.28**	.06	–			
8. Child inhibitory control 35 months	.07*	.12**	.17**	.13**	.18**	.02	.11**	–		
9. Child attention shifting 35 months	.07	.30**	.31**	.19**	.24**	.11**	.18**	.21**		–
*N*	1292	1292	1292	1204	1221	1063	787	912		842
Minimum	1	0	0	0.00	−2.33	−2.33	−1.44	−1.98		−1.98
Maximum	2	1	1	16.49	2.30	8.22	1.28	1.18		1.29
Mean	–	–	–	1.81	−0.02	0.00	−0.93	−0.25		−0.60
*SD*	–	–	–	1.68	0.85	2.02	0.66	0.69		0.78

*Note. SD* = standard deviation. Child sex: 1 = *male*, 2 = female. Child race: 0 = *African American*, 1 = *White*. (Residential) State: 0 = *North Carolina*, 1 = *Pennsylvania*.

* *p* < .05,

** *p* < .01.

**Table 2.** Results of the path model

	Working memory (*R*^2^ = .13)				Inhibitory control (*R*^2^ = .07)					Attention shifting (*R*^2^ = .13)		
35 months outcomes	*B*	*SE*	*t*	*β*	*B*	*SE*	*t*	*β*	*B*	*SE*	*t*	*β*
State	1.25	0.65	1.95	0.09	2.34	0.59	3.97***	0.17	3.14	0.71	4.43***	0.20
Child sex	0.93	0.44	2.15*	0.07	1.20	0.45	2.69**	0.09	1.04	0.51	2.05*	0.07
Child race	1.82	0.66	2.79**	0.14	−0.90	0.63	−1.43	−0.07	1.57	0.76	2.06*	0.10
Income-to-needs ratio	0.40	0.18	2.29*	0.10	0.23	0.13	1.77	0.06	0.13	0.17	0.72	0.03
M sensitivity	1.06	0.41	2.60**	0.14	0.59	0.37	1.57	0.07	1.37	0.43	3.17**	0.15
C negative reactivity	0.15	0.13	1.11	0.05	0.07	0.15	0.50	0.02	0.34	0.16	2.11*	0.09
C negative reactivity^2^	−0.01	0.06	−0.23	−0.01	0.00	0.04	−0.08	0.00	0.02	0.05	0.31	0.01
C NR × M sensitivity	−0.04	0.17	−0.24	−0.01	−0.23	0.18	−1.29	−0.06	0.08	0.19	0.38	0.02
C NR^2^ × M sensitivity	0.01	0.06	0.13	0.01	0.12	0.05	2.33*	0.12	−0.06	0.07	−0.89	−0.05

*Note*. C = *child*; M = *mother*; NR = *negative reactivity*; *SE* = standard error. Child sex: 1 = *male*, 2 = *female*. Child race: 0 = *African American*, 1 = *White*. (Residential) State: 0 = *North Carolina*, 1 = *Pennsylvania*.

* *p* < .05,

** *p* < .01,

*** *p* < .001.
