# Supplementary material for: A dynamic systems perspective towards executive function development: Susceptibility at both ends for inhibitory control
Source: Dev Psychopathol. Author manuscript; Available in PMC 2023 Nov 1. (PMC9437139; doi:10.1017/S0954579422000037)
Supplement: 1 [file NIHMS1770157-supplement-1.docx]

Supplemental Material

In the supplemental material, we used maternal sensitivity at 6 months alone in the path model to run the same analysis as in the main manuscript. This analytic model is more similar to those in Geeraerts et al. (2020)’s study, so findings can be comparable.

The path model had a perfect model fit due to being saturated, χ^2^(0) = 0.000, CFI = 1.000, TLI = 1.000, RMSEA = .000 (90% CI [.000, .000]). As shown in Table S1, maternal sensitivity at 6 months predicted higher attention shifting (*B* = 1.00, *SE* = 0.44, *t* = 2.29, *p* = .02) at 35 months. Additionally, an interaction between the quadratic term of infant negative reactivity and maternal sensitivity, both assessed at 6 months, emerged in predicting preschoolers *inhibitory control* (Figure S1). The U-shaped quadratic effect of infant negative reactivity on preschoolers’ inhibitory control was only significant when maternal sensitivity was high (*B* = 0.10, *SE* = 0.05, β = .10, *t* = 2.08, *p* = .04). When maternal sensitivity at 6 months was low, infant negative reactivity did not predict inhibitory control in quadratic (*B* = -0.08, *SE* = 0.07, β = -.07, *t* = -1.16, *p* = .25) or linear formats (*B* = 0.30, *SE* = 0.21, β = .09, *t* = 1.41, *p* = .16). The covariance of attention shifting and inhibitory control was correlated (*B* = 6.91, *SE* = 1.79, *t* = 3.86, *p* < .001), but not those between attention shifting and working memory (*B* = 2.67, *SE* = 1.66, *t* = 1.61, *p* = .11) or between inhibitory control and working memory (*B* = 1.91, *SE* = 1.47, *t* 1.30, *p* = .19).

There are a few minor differences between findings in the supplementary analysis and analysis in the main manuscript. First, maternal sensitivity at 6 months alone did not predicted higher working memory, but maternal sensitivity throughout early childhood did. Second, infant negative reactivity at 6 months did not predicted higher attention shifting, whereas in the main analysis this association was significant after controlling for maternal sensitivity throughout early childhood. Third, residential state became a significant predictor of working memory, and income-to-needs ratio became a significant predictor of higher inhibitory control, whereas in the main analysis both relations were just approaching significance after controlling for the effects of maternal sensitivity throughout early childhood.

Table S1.

*Results of the Path Model.*

| 35 months outcomes | Working Memory (R^2^ = .12) | | | | Inhibitory Control (R^2^ = .06) | | | | Attention Shifting (R^2^ = .13) | | | |
| --- | --- | --- | --- | --- | --- | --- | --- | --- | --- | --- | --- | --- |
|  | *B* | *SE* | *t* | *β* | *B* | *SE* | *t* | *β* | *B* | *SE* | *t* | *β* |
| State | 1.28 | 0.65 | 1.98* | 0.10 | 2.36 | 0.59 | 4.02*** | 0.17 | 3.17 | 0.72 | 4.43*** | 0.20 |
| Child sex | 0.94 | 0.44 | 2.16* | 0.07 | 1.19 | 0.45 | 2.64** | 0.09 | 1.03 | 0.51 | 2.00* | 0.07 |
| Child race and ethnicity | 2.03 | 0.65 | 3.14** | 0.15 | -0.76 | 0.63 | -1.22 | -0.06 | 1.71 | 0.76 | 2.27* | 0.11 |
| Income-to-needs ratio | 0.50 | 0.17 | 2.93** | 0.13 | 0.32 | 0.12 | 2.55* | 0.08 | 0.22 | 0.17 | 1.30 | 0.05 |
| M sensitivity | 0.63 | 0.45 | 1.40 | 0.08 | 0.40 | 0.38 | 1.04 | 0.05 | 1.00 | 0.44 | 2.29* | 0.10 |
| C negative reactivity | 0.56 | 0.53 | 1.06 | 0.17 | 0.91 | 0.56 | 1.64 | 0.27 | 0.23 | 0.64 | 0.35 | 0.06 |
| C negative reactivity^2^ | -0.10 | 0.24 | -0.42 | -0.10 | -0.32 | 0.17 | -1.86 | -0.30 | 0.03 | 0.19 | 0.13 | 0.02 |
| C NR × M sensitivity | -0.14 | 0.18 | -0.78 | -0.13 | -0.29 | 0.18 | -1.59 | -0.26 | 0.04 | 0.22 | 0.20 | 0.03 |
| C NR^2^ × M sensitivity | 0.03 | 0.08 | 0.40 | 0.09 | 0.12 | 0.05 | 2.15* | 0.33 | -0.01 | 0.06 | -0.13 | -0.02 |

*Note. SE =* standard error. C = child. M = mother. NR = negative reactivity. Child sex: 1 = male, 2 = female. Child race and ethnicity: 0 = African American, 1 = non-Hispanic White. (Residential) State: 0 = North Carolina, 1 = Pennsylvania.

* *p* < .05, ** *p* < .01, *** *p* < .001.

*

*Note.* Every unit of the x-axis indicates one standard deviation (*SD*), and zero indicate the mean of infant negative reactivity. The actual variable range is -1.15 – 4.07 *SD*.

*Figure 1.* The interaction between observed infant negative reactivity at 6 months and maternal sensitivity predicting child inhibitory control at 35 months.
